# Identification of the Causal Agent of Brown Leaf Spot on Kiwifruit and Its Sensitivity to Different Active Ingredients of Biological Fungicides

**DOI:** 10.3390/pathogens11060673

**Published:** 2022-06-10

**Authors:** Jia Chen, Fei Ran, Jinqiao Shi, Tingting Chen, Zhibo Zhao, Zhuzhu Zhang, Linan He, Wenzhi Li, Bingce Wang, Xuetang Chen, Weizhen Wang, Youhua Long

**Affiliations:** 1 Research Center for Engineering Technology of Kiwifruit, Institute of Crop Protection, College of Agriculture, Guizhou University, Guiyang 550025, China; c18184436145@126.com (J.C.); rf18786614232@126.com (F.R.); shijq163@163.com (J.S.); gzctt126@126.com (T.C.); zbzhao@gzu.edu.cn (Z.Z.); zhuzhuzhang9612@126.com (Z.Z.); gzhln9618@126.com (L.H.); lwz9512@126.com (W.L.); wbcgzu@126.com (B.W.); chenxt951231@126.com (X.C.); wzwang@gzu.edu.cn (W.W.); 2 Teaching Experimental Field of Guizhou University, Guizhou University, Guiyang 550025, China

**Keywords:** kiwifruit, brown leaf spot disease, *Fusarium graminearum*, biological characteristics, active ingredients of biological fungicides

## Abstract

Kiwifruit (*Actinidia chinensis*) is an important commercial crop in China, and the occurrence of diseases may cause significant economic loss in its production. In the present study, a new pathogen that causes brown leaf spot disease on kiwifruit was reported. The fungus was isolated from an infected sample and identified as *Fusarium graminearum* based on morphological and molecular evaluation. Koch’s postulates were confirmed when the pathogen was re-isolated from plants with artificially induced symptoms and identified as *F. graminearum*. Based on the biological characteristics of the pathogen, it was determined that: its optimal growth temperature was 25 °C; optimal pH was 7; most suitable carbon source was soluble starch; most suitable nitrogen source was yeast powder; and best photoperiod was 12 h light/12 h dark. Further investigations were conducted by determining 50% effective concentrations (EC_50_) of several active ingredients of biological fungicides against *F. graminearum*. The results showed that among the studied fungicides, tetramycin and honokiol had the highest antifungal activity against this pathogen. Our findings provide a scientific basis for the prevention and treatment of brown leaf spot disease on kiwifruit.

## 1. Introduction

Kiwifruit (*Actinidia chinensis*), a perennial deciduous woody liana, is native to China. In recent years, it has become an important commercial crop and is widely cultivated in New Zealand, Italy, China, and several other countries. It is favored by consumers worldwide because it contains more key micronutrients, such as potassium, calcium, and folic acid, than most other fruits [1]. According to the Chinese kiwifruit industry development report in 2020, the planting area of kiwifruit in China was 290,700 hm^2^ by the end of 2019, with the total output reaching 3,000,000 tons. Moreover, the area under plantation and kiwifruit output in China still rank first in the world. With the increasing kiwifruit planting area worldwide, fungal and bacterial diseases have been reported [2,3]. Fungal leaf spot diseases have become an increasingly serious threat to kiwifruit production in open-field orchards. Diseased plants are generally characterized by dark brown ring spots, grayish brown ring spots, silvering gray leaf blight, zonate leaf blight, and angular leaf spots [4]. Different fungal species, such as *Alternaria alternata*, *Nigrospora oryzae*, *Fusarium tricinctum*, *Colletotrichum* spp., *Diaporthe* spp., *Phoma* spp., and *Epicoccum* spp., have been reported as leaf spot pathogens [4,5,6,7], which cause severe loss in yield in the kiwifruit industry. 

*Fusarium graminearum* (Teleomorph *Gibberella zeae* Schw. Petch.), which belongs to ascomycetes, has been ranked fourth among the world’s top ten plant pathogenic fungi in terms of scientific and economic importance [8]. In general, *F. graminearum* is a causal pathogen of head blight or scab on barley (*Hordeum*), rice (*Oryza*), wheat (*Triticum*), and maize (*Zea*) stalk rot, as well as of spoilage on water bamboo shoots [9,10]. Its spores spread by wind or rain in the spring season and infect plants at optimal temperatures [11,12]. In addition to limiting healthy crop development, *F. graminearum* produces a variety of mycotoxins that pose safety risks to human and animal health [13]. Earlier studies have shown that multiple factors, such as temperature, pH, light, carbon source, and nitrogen source, have significant influences on pathogen growth and pathogenicity [14,15,16,17]. Therefore, it is necessary to carry out a study of the biological characteristics of the pathogen to investigate the suitable conditions for its occurrence and prevalence. Synthetic fungicides, such as metconazole [18] and carbendazim [19], are commonly used to control *F. graminearum* infection. However, biological control is becoming an important strategy in controlling *F. graminearum* to ensure food safety [20]. 

In July 2019, typical brown leaf spots were observed in ‘Guichang’ kiwifruit orchards (*Actinidia deliciosa* cv. ‘Guichang’) in Xifeng County, Guizhou Province, China. The disease initially appeared as a few small brown spots in the early stage and gradually expanded into larger lesions. The disease incidence rate in 16 kiwifruit orchards (20 hm^2^) was 21%, resulting in a large number of fallen leaves and approximately 9–16% of yield loss per orchard. It was determined that *F. graminearum* was the causal agent of brown leaf spot on kiwifruit in this planting area, and this was the first time that it was reported in the area. The isolated pathogen was used for further investigations by exploring its biological characteristics and determining its sensitivity to different active ingredients of biological fungicides.

## 2. Results

### 2.1. Pathogenicity Test of the Isolated Strains

**Figure 1:**
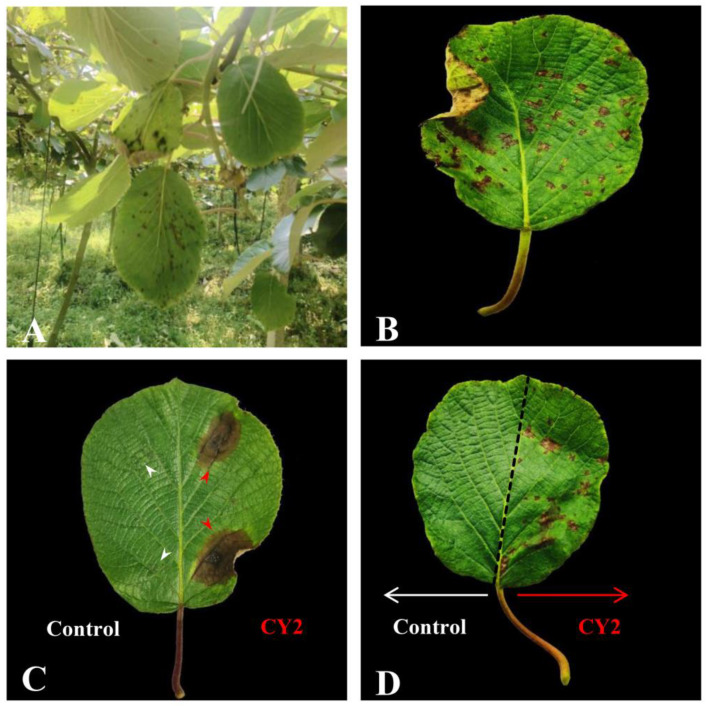
(**A**,**B**) Natural field symptoms of brown leaf spot on kiwifruit. (**C**) In the wounded inoculation method, six days after inoculation with isolate CY2 spore suspension, the artificially inoculated leaves showed brown spots similar to those observed in the orchard, while the control inoculation did not show brown spots (white and red arrows indicate the sites of inoculation). The experiment was repeated three times with similar results. (**D**) On the ninth day after inoculation with isolate CY2 spore suspension, the artificially inoculated leaves showed brown spots, while no symptoms were observed on the control inoculation (the dashed line indicates the major vein, white and red arrows indicate different inoculation treatments). The experiment was repeated three times with similar results.

Typical brown leaf spots were observed in the ‘Guichang’ kiwifruit orchards in July 2019. They appeared as a few small brown spots in the early stage and gradually expanded into larger lesions (Figure 1A,B). From the infected samples collected, a total of 37 fungal isolates were obtained. A pathogenicity test was conducted on these isolates using wounded and unwounded inoculation methods. The results showed that only isolate CY2 was pathogenic to kiwifruit. In the wounded inoculation method, the artificially inoculated leaves showed similar brown spots as those in the orchard six days after inoculation with isolate CY2 spore suspension, whereas the control inoculation did not show any brown spots (Figure 1C). In the unwounded inoculation method, the artificially inoculated leaves showed brown spots on the ninth day after inoculation with isolate CY2 spore suspension, whereas the control inoculation did not show any brown spots (Figure 1D). A morphologically similar fungus was re-isolated from the lesions of the artificially inoculated leaves. The results of the three trials were similar, which proved that the highly pathogenic isolate CY2 caused brown leaf spot disease on kiwifruit.

### 2.2. Identification of Isolate CY2

**Figure 2:**
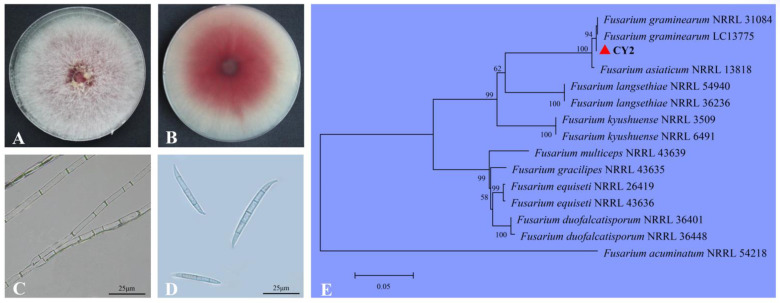
(**A**,**B**) Colony morphology of isolate CY2 on potato dextrose agar (PDA) after six days of cultivation. (**C**) Mycelia of isolate CY2 on PDA, with branches and septa. (**D**) Macroconidia produced on CLA with 5–6 septa. (**E**) The evolutionary history of the investigated *Fusarium graminearum* isolate CY2 (indicated by bold letters) inferred by the neighbor-joining method (1000 bootstraps for confidence level) based on the combined ITS, *TEF-1α*, and *RPB2* genes.

After culturing at 25 °C for six days, the colony of the highly pathogenic isolate CY2 grew rapidly on potato dextrose agar (PDA; Beijing Land Bridge Technology Co., Ltd., Beijing, China) and produced a large number of dense hyphae with red pigments (Figure 2A,B), and the mycelium had branches and septa (Figure 2C). Macroconidia of fungi cultured on carnation leaf-piece agar (CLA; 4 pieces of 5 mm long sterile carnation leaves, 10 mL of 2% water agar) had 3–5 septa, which were slender, sickle-shaped to nearly straight, with an average size of 26.4–56.7 × 3.3–7.5 μm (n = 50; Figure 2D). The isolate CY2 was identified as *F. graminearum* based on these morphological characteristics [10,21].

For molecular identification, different fragments of this isolate were amplified and sequences were deposited in GenBank. The sequence of the ITS region (MW871547) had 100% identity to sequences of several species stored in GenBank, including *F. graminearum*. The *TEF-1α* sequence (MW876479) was 100% homologous to that of *F. graminearum* (MN381089), while the *RPB2* sequence (MW876480) showed 99.89% similarity to that of *F. graminearum* (MW233447). A phylogenetic tree was constructed using ITS, *TEF1-α*, and *RPB2* gene sequences of isolate CY2, along with a few reference isolates obtained from GenBank (Table 1). It showed that isolate CY2 clustered together with other strains of *F. graminearum* obtained from GenBank. Therefore, results of the molecular analysis supported our morphological identification, which demonstrated that the highly pathogenic isolate CY2 was *F. graminearum* (Figure 2E).

### 2.3. Biological Characteristics of Isolate CY2

**Figure 3:**
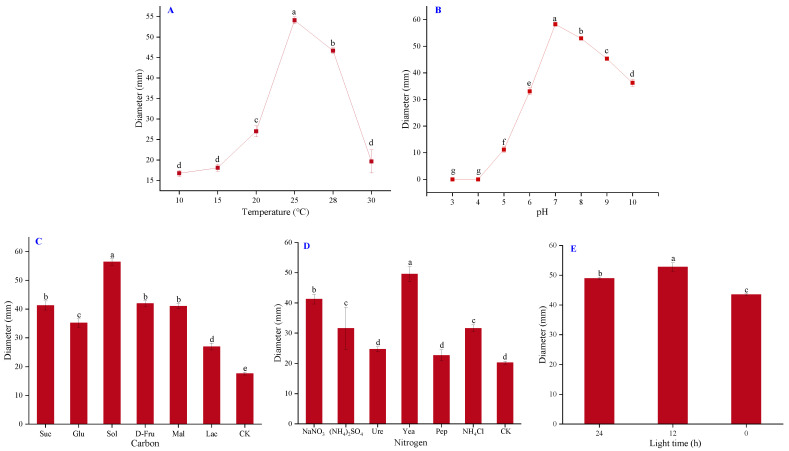
Mycelial diameters under different (**A**) temperatures, (**B**) pH, (**C**) carbon sources, (**D**) nitrogen sources, and (**E**) light time conditions. The diameters were determined after four days of cultivation of *F*. *graminearum* isolate CY2. The error bar indicates standard deviations (SD), each value is the mean ± SD of three replicates, and different lower-case letters represent significant differences at the 5% level (*p* < 0.05).

After four days of culture at the temperature range of 10–30 °C, the colony diameter was 16.75 mm at 10 °C, 18.08 mm at 15 °C, 27 mm at 20 °C, 54.08 mm at 25 °C, 46.67 mm at 28 °C, and 19.67 mm at 30 °C (Figure 3A). The results showed that 25 °C was the optimal temperature for the growth of isolate CY2. Furthermore, the isolate grew at the range of pH 5–10, and its growth ceased at pH 3–4. The mycelial growth peak appeared at pH 7 with a colony diameter of 58.25 mm, showing that a moderate pH environment was optimal for its growth (Figure 3B). Soluble starch (Sol) was the optimal carbon source and yeast powder (Yea) was the optimal nitrogen source, as they resulted in the largest colony diameters among the treatments (Figure 3C,D). Regarding the light requirements, isolate CY2 could grow in all light conditions, but a daily 12 h photoperiod was the most beneficial for its growth (Figure 3E).

### 2.4. Toxicity Effects of Several Active Ingredients of Biological Fungicides against F. graminearum CY2

**Figure 4:**
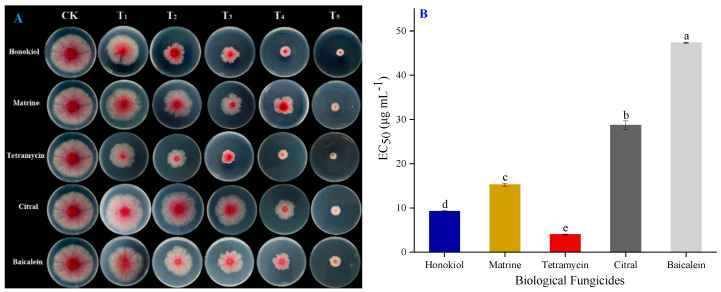
(**A**) Mycelial growth inhibition of *Fusarium graminearum* isolate CY2 after the application of different active ingredients of biological fungicides under a series of concentrations, with fungicide free plates (CK) as the control. (**B**) EC_50_ value of different fungicides applied to isolate CY2. The error bar indicates standard deviations (SD), each value is the mean ± SD of three replicates, and different lower-case letters represent significant differences at the 5% level (*p* < 0.05).

Screening active ingredients of biological fungicides against isolate CY2 is helpful for developing environmentally safe alternatives for the control of *F. graminearum*. In the present study, the sensitivity of isolate CY2 to five different active ingredients of biological fungicides, including honokiol, matrine, tetramycin, citral, and baicalein, was determined in vitro, (Figure 4 and Table 2). Positive slopes in the regression equations indicated a positive correlation between concentration and growth inhibition and *R^2^* values close to 1 indicated that all equations were reliable. Tetramycin had the lowest EC_50_ value of 4.02 ± 0.05 μg mL^−1^, followed by honokiol and matrine, with EC_50_ values of 9.26 ± 0.11 and 15.2 ± 0.31 μg mL^−1^, respectively. Citral and baicalein showed relatively weak inhibition effects, with EC_50_ values of 28.6 ± 0.99 and 47.3 ± 0.12 μg mL^−1^, respectively. This indicated that tetramycin and honokiol had the highest antifungal activity against *F. graminearum*, while baicalein had the lowest.

## 3. Discussion

In the present study, although a total of 37 fungal isolates were obtained from the infected kiwifruit samples with symptoms of brown leaf spot, only isolate CY2 was confirmed to be a real pathogen based on Koch’s postulates [22]. Using wounded and unwounded inoculation methods, we showed that the other isolates were not pathogenic to kiwifruit, indicating they were probably saprophytic fungi on kiwifruit leaves. It is also possible that some of these fungi are pathogens, but indoor conditions were not suitable for their infection. Our morphological and molecular identification methods suggested that the pathogenic isolate CY2 was *F. graminearum*. The infection of kiwifruit with *Fusarium* species has led to concerning problems in its agricultural production [7,23,24,25,26]. However, this is the first report of the pathogen *F. graminearum* causing brown leaf spot on kiwifruit. High humidity is favorable to the distribution and survival of different pathogens, including *F. graminearum* [27], which may be an important reason why isolate CY2 was highly pathogenic to kiwifruit in the pathogenicity assays, as they were conducted under high humidity conditions. *F. graminearum* produces both ascospores and macroconidia, which can be transmitted by air, rain, and insects [11,28,29]. Most previous studies have used macroconidia for inoculation, since they are more readily produced than ascospores [30,31,32,33,34].

To determine the conditions suitable for the occurrence of different pathogens, it is essential to investigate their biological characteristics [35,36]. The findings of previous studies on the biological characteristics of *F. graminearum* were similar to ours, indicating that the optimal temperature for *F. graminearum* mycelial growth is 25 °C; optimal pH is 7; most suitable nitrogen source is Yea; and most suitable photoperiod is 12 h light/12 h darkness [10,37]. However, some findings of the present study differed from those of previous studies. For example, a previous study found that the most suitable carbon source for *F. graminearum* was glucose (Glu), while ours showed that it was Sol [10]. The differences in biological characteristics may be caused by differences in strains and regions that were evaluated in the study.

In recent years, food safety has become an important topic, and synthetic fungicides have been shown to cause serious health problems in humans [38,39,40]. Moreover, *F. graminearum* has developed resistance to synthetic fungicides, such as azoles and carbendazim, because of their long-term usage [41,42,43,44,45]. Therefore, it is of great significance to develop active ingredients of biological fungicides to combat this critical pathogen. In the present study, the sensitivity of *F. graminearum* CY2 to five active ingredients of biological fungicides was determined, and we found that among them, tetramycin and honokiol showed the highest inhibitory activities against this isolate. Tetramycin is a polyene macrolide antibiotic produced by *Streptomyces ahygroscopicus* var. *wuzhouensis*, and it has a strong antimicrobial effect and can enhance host disease resistance by inducing the activity of defense enzymes [46]. Honokiol is a polyphenolic compound obtained from *Magnolia officinalis* and can inhibit fungal mycelial growth by inducing apoptosis and autophagy [47]. These two active ingredients of biological fungicides displayed excellent inhibitory activities against *F. graminearum* isolate CY2 in vitro, suggesting that they are potential environmentally safe fungicides for treating brown leaf spot disease caused by *F. graminearum* in kiwifruit. However, their control efficacies against brown leaf spot disease in the field should be further investigated. Specifically, their mechanisms of antifungal action remain unknown and require further study.

## 4. Materials and Methods

### 4.1. Sampling, Isolation, and Purification

Leaf samples were collected from kiwifruit plants in the ‘Guichang’ kiwifruit orchards (*Actinidia deliciosa* cv. ‘Guichang’) in Xifeng County, Guizhou Province, China (27°3′15.74″ N, 106°31′13.9″ E). Infected leaf tissue was cut into small pieces (5 mm × 5 mm), and their surfaces were disinfected with 75% ethanol and washed with sterile distilled water thrice. The cleaned tissue samples were dried on sterile absorbent paper and then transferred onto PDA plates. The inoculated plates were incubated at 25 °C in a growth chamber for four days. The emerging fungal hyphae were transferred to fresh PDA plates for further culture. The obtained isolates were preserved in 20% (*v*/*v*) glycerin at −20 °C.

### 4.2. Pathogenicity Tests

Testing pathogenicity according to Koch’s postulates [48], the fungal isolates with various colony morphologies were cultivated on PDA (for most isolates) at 25 °C for six days. For isolate CY2, the CLA Petri plate was used, as *F. graminearum* could easily produce spores on this medium [21]. The conidia were detached using a sterilized glass sprayer, and the conidial concentration was adjusted to 1 × 10^6^ conidia mL^−1^ using a cytometer (Solarbio Science and Technology Co., Ltd., Beijing, China). Healthy kiwifruit leaves (cv. ‘Guichang’) were surface disinfected in 0.1% NaOCl, washed with water, and air dried. Two methods were used for inoculation: wounded and unwounded inoculation [23,24,25]. For wounded inoculation, kiwi leaves were pierced three times with a sterile needle, after which the pierced sites were inoculated with 5 μL of spore suspension or the same amount of sterile water (control). For unwounded inoculation, 500 μL of spore suspension was evenly sprayed on healthy kiwi leaves on the right side of the major vein, and 500 μL of sterile water was sprayed on the left side (control). All leaves with petioles were wrapped in wet cotton to retain moisture. Three replicates of each treatment were performed to assess the pathogenicity of each isolate, and the pathogenicity assay was repeated three times. The artificially inoculated leaves were cultivated in a growth chamber at 25 °C with 90% relative humidity and a light time of 12 h per day. The pathogen was re-isolated from the lesion area of inoculated leaves that developed symptoms and identified morphologically.

### 4.3. Morphological Characterization

The purified pathogenic fungus discs (d = 6 mm) were inoculated on PDA for mycelial growth and on CLA for macroconidium production. After culturing at 25 °C for six days, the culture characteristics of hyphae and macroconidia were observed and recorded using a binocular microscope (Leica DM500, Leica Microsystems (Shanghai) Trading Co., Ltd., Shanghai, China).

### 4.4. DNA Extraction, PCR Amplification, Sequencing, and Phylogenetic Analysis

After the culture at 25 °C for six days, the mycelia were collected and freeze dried in a vacuum freeze-dryer (LGJ-10E, Beijing Sihuan Scientific Instrument Factory Co., Ltd., Beijing, China). Genomic DNA was extracted using the Ezup Column Fungal Genomic DNA Extraction Kit (Sangon Bioengineering Ltd., Shanghai, China). The primers ITS1/ITS4 [49], EF1-728F/EF1-986R [50], and RPB2-5F2/fRPB2-7cR [51,52] were used to amplify the ribosomal DNA internal transcribed spacer (ITS) region, translation elongation factor 1-alpha encoding gene (*TEF-1α*), and the second largest subunit of RNA polymerase II encoding gene (*RPB2*) genes, respectively (Table 3). Polymerase chain reaction (PCR) amplification was carried out in a Bio-Rad T100^TM^ Thermal Cycler (Bio-Rad Laboratories Co., Ltd., Shanghai, China) in a 20 μL reaction mixture comprising 10 μL of 2×Taq PCR StarMix with a loading dye (Sangon, Inc., Shanghai, China), 1 μL of the DNA template, 1 μL of each primer, and 7 μL of ddH_2_O. The PCR conditions were as follows: initial denaturation at 94 °C for 2 min, 35 cycles of amplification (denaturation at 94 °C for 30 s, annealing at 60 °C for 30 s, and extension at 72 °C for 60 s), and a final extension at 72 °C for 10 min. Sequencing of the PCR products was conducted by Sangon Bioengineering Ltd. (Shanghai, China), and the BLAST network service was used to compare the sequences with other sequences deposited in GenBank for similarity analysis.

Reference sequences of species closely related to the isolated pathogens were obtained from GenBank for the construction of an ITS–*TEF-1α*–*RPB2* phylogenetic tree. Multiple sequence comparison by log expectation (MUSCLE) was used to perform sequence alignments. Phylogenetic relationships were determined by MEGA v7.0 (Mega Limited Inc., Auckland, New Zealand) using the neighbor-joining method (bootstrap analysis with 1000 replicates), and *Fusarium acuminatum* was used as an outgroup [53,54].

### 4.5. Biological Characteristics of Isolate CY2

The biological characteristics of the isolate were investigated, as described in previous studies, by evaluating the effects of temperature, pH, carbon source, nitrogen source, and light on the mycelial growth of the fungal pathogen [2,10,55]. The pathogen was cultured on PDA medium in darkness at 10–30 °C to determine the optimum culture temperature and at pH 3–10 to determine the optimal pH value. Czapek Dox agar (CDA; Beijing Land Bridge Technology Co., Ltd., Beijing, China) containing equal carbon quantity of (Glu, Sol, d-fructose (D-Fru), maltose (Mal), and lactose (Lac) instead of sucrose (Suc) was used to determine the optimal carbon source, and the medium with no carbon source was used as blank control (CK); and CDA containing equal nitrogen quantity of (NH_4_)_2_SO_4_, urea (Ure), Yea, peptone (Pep), and NH_4_Cl instead of NaNO_3_ were used to determine the optimal nitrogen source, while the medium with no nitrogen source was used as blank control (CK). The pathogen was cultured on PDA at a photoperiod of 24 h light, 24 h darkness, or 12 h light/12 h darkness to determine the influence of light time on its growth. For each evaluation, the pathogen was cultivated on PDA Petri dishes (d = 9 cm) before transferring the mycelial discs (d = 6 mm) from fungal colonies to the center of the plates with three replicates. Except for the temperature and light treatment, all other treatments were cultured in darkness at 25 °C for four days, and colony diameter was measured in two vertical directions.

### 4.6. In Vitro Toxicity Tests

In vitro toxicity of several active ingredients of biological fungicides against the isolate CY2 was evaluated according to Xing’s method [56]. Active ingredients of biological fungicides were dissolved in organic solvent *N*,*N*–dimethylformamide (DMF) to prepare stock solutions, which were then diluted with water and added into the PDA medium to obtain different concentrations (Table 4). Fungicide-free PDA was used as control. Mycelial discs (d = 6 mm) from the periphery of the colonies were inoculated at the center of the plate. After incubation at 25 °C for five days, mycelial diameters were measured in two vertical directions. Three replicates were evaluated for each treatment, and one plate was randomly selected from each treatment and photographed using a digital camera (ZV-1, Sony Co., Ltd., Beijing, China). The growth inhibition rate was calculated according to the following Formula (1):

Growth inhibition rate = 100% × (Dc − Dt)/(Dc − 6)(1)

where Dc is the diameter of the colony on the control plate, 6 mm is the diameter of inoculated mycelial discs, and Dt is the diameter of the colony grown on plates with different concentrations of active ingredients of biological fungicides. The growth inhibition rate of each treatment concentration was converted into a probability value, and the concentration was converted into a logarithmic value. The toxicity regression equation, determination coefficient (*R*^2^), and EC_50_ values of each treatment were calculated using the DPS data processing system [57].

### 4.7. Data Analysis

The data were analyzed using Microsoft Excel 2010 (Microsoft Inc., Redmond, WA, USA) and visualized using Origin v7.0 (Origin Lab Corporation, Northampton, MA, USA) [58]. One-way analysis of variance (ANOVA) was conducted with DPS v16.0 (Ruifeng Information Technology Co., Ltd., Zhejiang, China), and Duncan’s multi-range test was used to determine statistical significance at *p* < 0.05 [57].

## 5. Conclusions

In this study, we aimed to determine the causal agent of brown leaf spot disease observed in kiwifruit orchards in Guizhou. Based on Koch’s postulates, morphological identification, and molecular characteristics, *F. graminearum* was identified as the fungal pathogen causing this disease in kiwifruit. To the best of our knowledge, this is the first study to report that brown leaf spot on kiwifruit was caused by *F. graminearum*. Furthermore, the biological characteristics of *F. graminearum* CY2 were investigated, and the inhibitory effects of several active ingredients of biological fungicides on its growth were evaluated. Our findings are significant for the prevention and control of this important pathogen.

## Figures and Tables

**Table 1:** Reference isolates used in the present study and their GenBank accession numbers.

Species	Culture Accession	GenBank Accession		
		ITS	*TEF-1α*	*RPB2*
*Fusarium graminearum*	NRRL 31084	-	MW233103	MW233447
*Fusarium graminearum*	LC 13775	-	MW620072	MW474597
*Fusarium asiaticum*	NRRL 13818	-	MW233069	MW233412
*Fusarium acuminatum*	NRRL 54218	HM068326	HM068316	HM068336
*Fusarium equiseti*	NRRL 26419	GQ505688	GQ505599	GQ505777
*Fusarium equiseti*	NRRL 43636	GQ505752	GQ505663	GQ505841
*Fusarium langsethiae*	NRRL 54940	-	MW233138	MW233482
*Fusarium langsethiae*	CBS 36236	-	MW233114	MW233458
*Fusarium duofalcatisporum*	NRRL 36401	-	GQ505651	GQ505829
*Fusarium duofalcatisporum*	NRRL 36448	GQ505741	GQ505652	GQ505830
*Fusarium kyushuense*	NRRL 3509	NR152943	MW233056	MW233399
*Fusarium kyushuense*	NRRL 6491	-	MW233057	MW233400
*Fusarium multiceps*	NRRL 43639	GQ505755	GQ505666	GQ505844
*Fusarium gracilipes*	NRRL 43635	GQ505751	GQ505662	GQ505840

**Table 2:** Toxicities of different active ingredients of biological fungicides against *Fusarium graminearum* CY2.

Active Ingredients of Biological Fungicides	Regression Equation	Determination Coefficient (*R*^2^)	EC_50_ (μg mL^−1^)	95% Confidence Interval
98% Honokiol DP	Y = 3.4834 + 1.5742x	0.9876	9.26 ± 0.11	7.97–10.60
98% Matrine DP	Y = 3.1989 + 1.5251x	0.9831	15.2 ± 0.31	13.11–17.54
1.5% Tetramycin AS	Y = 4.3763 + 1.0306x	0.9817	4.02 ± 0.05	3.10–5.23
97% Citral AS	Y = 2.1413 + 1.974x	0.9825	28.6 ± 0.99	23.99–32.84
98% Baicalein DP	Y = 2.457 + 1.5172x	0.9730	47.3 ± 0.12	39.31–57.23

Each value indicates the mean ± SD of three replicates; X and Y represent active ingredients of biological fungicides concentration and growth inhibition rate, respectively.

**Table 3:** Primers used in the present study.

Target Region/Gene	Description	Primer	Sequence 5′ → 3′	Reference
ITS	Region with ribosomal RNA genes and two internal transcribed spacers	ITS1	TCCGTAGGTGAACCTGCGG	[49]
		ITS4	TCCTCCGCTTATTGATATGC	
*TEF-1α*	Translation elongation factor 1-α gene	EF1-728F	CATCGAGAAGTTCGAGAAGG	[50]
		EF1-986R	TACTTGAAGGAACCCTTACC	
*RPB2*	Second largest subunit of RNA polymerase II	fRPB2-7cR	CCCATRGCTTGTYYRCCCAT	[51]
		RPB2-5F2	GGGGWGAYCAGAAGAAGGC	[52]

**Table 4:** Concentration gradient of different active ingredients of biological fungicides used for treating isolate CY2.

Active Ingredients of Biological Fungicides	Manufacturer	Concentration Gradient (μg mL^−1^)				
		T_1_	T_2_	T_3_	T_4_	T_5_
98% Honokiol DP	Shanghai Macklin Biochemical Co., Ltd., Shanghai, China	4	8	16	32	64
98% Matrine DP	Aladdin Industrial Corporation, Shanghai, China	4	8	16	32	64
1.5% Tetramycin AS	Liaoning Wkioc Bioengineering Co., Ltd., Shenyang, China	4	8	16	32	64
97% Citral AS	Aladdin Industrial Corporation, Shanghai, China	5	10	20	40	80
98% Baicalein DP	Shanghai Macklin Biochemical Co., Ltd., Shanghai, China	10	20	40	80	160

DP, dust powder; AS, aqueous solution.

## Data Availability

The datasets generated and/or analyzed during the study are available from the corresponding author upon reasonable request.
