# Correction: Chen et al. Identification of the Causal Agent of Brown Leaf Spot on Kiwifruit and Its Sensitivity to Different Active Ingredients of Biological Fungicides. *Pathogens* 2022, *11*, 673

**DOI:** 10.3390/pathogens12111327

**Published:** 2023-11-08

**Authors:** Jia Chen, Fei Ran, Jinqiao Shi, Tingting Chen, Zhibo Zhao, Zhuzhu Zhang, Linan He, Wenzhi Li, Bingce Wang, Xuetang Chen, Weizhen Wang, Youhua Long

**Affiliations:** 1 Research Center for Engineering Technology of Kiwifruit, Institute of Crop Protection, College of Agriculture, Guizhou University, Guiyang 550025, China; c18184436145@126.com (J.C.); rf18786614232@126.com (F.R.); shijq163@163.com (J.S.); gzctt126@126.com (T.C.); zbzhao@gzu.edu.cn (Z.Z.); zhuzhuzhang9612@126.com (Z.Z.); gzhln9618@126.com (L.H.); lwz9512@126.com (W.L.); wbcgzu@126.com (B.W.); chenxt951231@126.com (X.C.); wzwang@gzu.edu.cn (W.W.); 2 Teaching Experimental Field of Guizhou University, Guizhou University, Guiyang 550025, China

## Error in Figure/Table

In the original publication [1], there was a mistake in Figure 4A and Table 3 as published. The original Figure 4A incorrectly included two identical plates. The corrected Figure 4 is shown below. The reference numbers in the original Table 3 are not correct. The correct reference numbers should be [49–52], instead of [26–29]. The corrected Table 3 is shown below. 

The authors apologize for any inconvenience caused and state that the scientific conclusions are unaffected. This correction was approved by the Academic Editor. The original publication has also been updated.

## Figures and Tables

**Figure 4:**
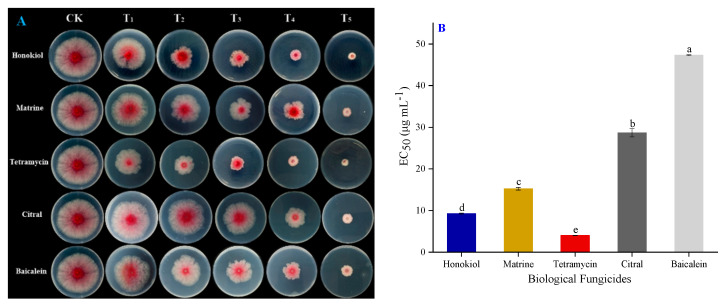
(**A**) Mycelial growth inhibition of *Fusarium graminearum* isolate CY2 after the application of different active ingredients of biological fungicides under a series of concentrations, with fungicide free plates (CK) as the control. (**B**) EC_50_ value of different fungicides applied to isolate CY2. The error bar indicates standard deviations (SD), each value is the mean ± SD of three replicates, and different lower-case letters represent significant differences at the 5% level (*p* < 0.05).

**Table 3:** Primers used in the present study.

Target Region/Gene	Description	Primer	Sequence 5′→3′	Reference
ITS	Region with ribosomal RNA genes and two internal transcribed spacers	ITS1	TCCGTAGGTGAACCTGCGG	[49]
		ITS4	TCCTCCGCTTATTGATATGC	
*TEF-1α*	Translation elongation factor 1-α gene	EF1-728F	CATCGAGAAGTTCGAGAAGG	[50]
		EF1-986R	TACTTGAAGGAACCCTTACC	
*RPB2*	Second largest subunit of RNA polymerase II	fRPB2-7cR	CCCATRGCTTGTYYRCCCAT	[51]
		RPB2-5F2	GGGGWGAYCAGAAGAAGGC	[52]

## References

[1] Chen J., Ran F., Shi J., Chen T., Zhao Z., Zhang Z., He L., Li W., Wang B., Chen X. (2022). Identification of the Causal Agent of Brown Leaf Spot on Kiwifruit and Its Sensitivity to Different Active Ingredients of Biological Fungicides. Pathogens.

